# Potent Immunity to Low Doses of Influenza Vaccine by Probabilistic Guided Micro-Targeted Skin Delivery in a Mouse Model

**DOI:** 10.1371/journal.pone.0010266

**Published:** 2010-04-21

**Authors:** Germain J. P. Fernando, Xianfeng Chen, Tarl W. Prow, Michael L. Crichton, Emily J. Fairmaid, Michael S. Roberts, Ian H. Frazer, Lorena E. Brown, Mark A. F. Kendall

**Affiliations:** 1 Delivery of Drugs and Genes Group (D2G2), Australian Institute for Bioengineering & Nanotechnology, The University of Queensland, Brisbane, Queensland, Australia; 2 Therapeutics Research Unit, School of Medicine, The University of Queensland, Brisbane, Queensland, Australia; 3 Diamantina Institute for Cancer, Immunology & Metabolic Medicine, The University of Queensland, Brisbane, Queensland, Australia; 4 Department of Microbiology & Immunology, The University of Melbourne, Parkville, Victoria, Australia; Veterinary Laboratories Agency, United Kingdom

## Abstract

**Background:**

Over 14 million people die each year from infectious diseases despite extensive vaccine use [1]. The needle and syringe—first invented in 1853—is still the primary delivery device, injecting liquid vaccine into muscle. Vaccines could be far more effective if they were precisely delivered into the narrow layer just beneath the skin surface that contains a much higher density of potent antigen-presenting cells (APCs) essential to generate a protective immune response. We hypothesized that successful vaccination could be achieved this way with far lower antigen doses than required by the needle and syringe.

**Methodology/Principal Findings:**

To meet this objective, using a probability-based theoretical analysis for targeting skin APCs, we designed the Nanopatch™, which contains an array of densely packed projections (21025/cm^2^) invisible to the human eye (110 µm in length, tapering to tips with a sharpness of <1000 nm), that are dry-coated with vaccine and applied to the skin for two minutes. Here we show that the Nanopatches deliver a seasonal influenza vaccine (Fluvax® 2008) to directly contact thousands of APCs, in excellent agreement with theoretical prediction. By physically targeting vaccine directly to these cells we induced protective levels of functional antibody responses in mice and also protection against an influenza virus challenge that are comparable to the vaccine delivered intramuscularly with the needle and syringe—but with less than 1/100^th^ of the delivered antigen.

**Conclusions/Significance:**

Our results represent a marked improvement—an order of magnitude greater than reported by others—for injected doses administered by other delivery methods, without reliance on an added adjuvant, and with only a single vaccination. This study provides a proven mathematical/engineering delivery device template for extension into human studies—and we speculate that successful translation of these findings into humans could uniquely assist with problems of vaccine shortages and distribution—together with alleviating fear of the needle and the need for trained practitioners to administer vaccine, e.g., during an influenza pandemic.

## Introduction

**Figure 1:**
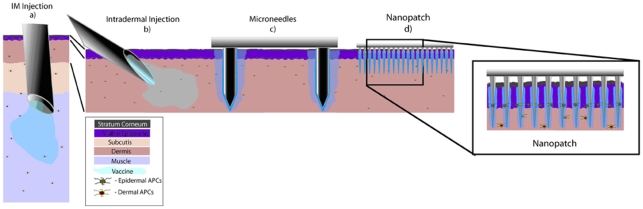
The concept of targeting antigen directly to skin antigen presenting cells (APCs) (in the epidermis and dermis) using Nanopatches™ – compared to existing needle-based delivery methods. Drawn to scale is the structure of human skin (which is thicker than mouse skin; including the location of Langerhans cells and dermal APCs). We also show, to scale, the geometry of different needle-based delivery devices (**a–c**) and the Nanopatch™ (**d**). (**a**) Intramuscular (IM) route directly inject a vaccine into muscle, which contains a low density of antigen presenting cells. (**b**) Intradermal (ID) injection delivers vaccine to the dermis of the skin, where there is an extensive network of resident professional APCs. These APCs can capture antigen, migrate to the draining lymph node, and orchestrate potent systemic immune responses. Therefore, ID delivery can achieve comparable immune responses to IM, but at about one-tenth of the vaccine dose. However, it is technically challenging to do an ID injection. (**c**) Microneedle techniques use sparsely packed needles with dimensions of hundreds of micrometers to deliver a liquid or dry form of the vaccine to the skin. This method is technically easier and is as efficient as ID injection. (**d**) The Nanopatch™ technique uses a very small and densely packed array (over 20,000 projections/cm^2^) to directly deposit vaccine material into the immediate vicinity of a large population of APCs in a small area. Therefore, numerous APCs can be directly involved in raising an immune response.

Vaccines can be more effective if they are delivered into the narrow layer just beneath the skin surface that contains a high density of antigen presenting cells (APCs) required to generate an immune response [2]–[4], rather than into the muscle where such cells present at a much lower density (**Fig 1**). One key focus for improvement is in achieving successful vaccination using the lowest dose possible. This is particularly important in the context of a rapidly emerging disease, such as pandemic influenza, where existing vaccination production methods are slow to meet the demand for population protection [5]. Some success in tackling this problem has been achieved by supplementing the vaccine with an adjuvant – although in many cases with an increase of adverse reactions [6]. Alternatively, targeting of vaccines directly to large populations of skin immune cells holds great potential in achieving protection, with significant dose sparing and improved tolerability profiles.

So far, however, only limited dose sparing gains have been achieved: delivery approaches vaccinating with conventional antigens have yielded functional disease protection, but with dose sparing gains of one order of magnitude compared to intramuscular injections without the use of an adjuvant. Most studies make use of a needle or microneedle(s) to deliver the vaccine to different tissue sites; and we illustrate these schematically in **Fig. 1 b–c**. It is compared to the current convention of most vaccines being delivered with a needle into muscle (**Fig 1a**). The closest alternative to this – and the most tested [7]– is delivering liquid vaccine with a fine needle to the dermis (**Fig 1b**); with, for example, influenza vaccines requiring one tenth of the antigen for a comparable immune response as standard intramuscular injection [3]. With a thinner target area, intradermal injection is technically difficult to administer. Recently, this difficulty has been mitigated by a more controlled fixed-penetration injection mechanism [8] (>1mm in length) – with a finer needle (∼100 µm in diameter) – while still yielding similar immunological results that are functional in the protection against disease (some work was also presented with even greater dose-sparing than 1∶10, but this is limited to total antibody readouts – which do not yield insights into protective efficacy against a particular disease). However, needle-based intradermal vaccine approaches pierce through the tightly-defined epidermal immune cell populations [2] (abundant in a class of APCs called Langerhans cells), delivering liquid vaccine as a single bolus into the dermis (**Fig 1b**) [2]. This may not be the most effective way of targeting antigens to the skin immune system: the epidermal immune cells are largely missed; and further below, in the dermis, the ‘pooled’ vaccine reaches dermal APCs, with much of it in the surrounding region away from these cells – and not directly accessible to them. By increasing the number of similarly shaped needles up to 5 – to deliver either liquid or solid-coated vaccine (e.g. **Fig 1c**) – vaccine interaction with the skin target cell populations is not fundamentally different; and therefore the resultant immune responses are also similar to individual needle injection to the dermis [4], [9], [10]. And by further increasing the number of micro-needles in an array to 190 on a 1 cm^2^ patch (then extended to 1314 microneedles on a 2 cm^2^ patch) [11], [12], better immune responses have been achieved following chicken egg albumin delivery (a non pathogenic protein; functional protection against a disease has therefore not been tested) in comparison with intramuscular injection. However, the induced immune responses are no higher than achieved by needle intradermal delivery. And dose-sparing effects were not explored [11].

Recently, other methods of vaccine delivery moving completely away from the needle (e.g., diffusion/permeation delivery [13], liquid jet injection [14], and ballistic microparticle injection [15]) have been pursued to meet these delivery challenges. However, their ability to consistently and directly deliver vaccines directly to a high population of skin immune cells with minimal cell damage is limited (resulting in comparable dose sparing gains as needle or microneedle-based systems). Also, these devices are hindered by practical considerations, such as size, expense of manufacture or complexity of operation

Here, we present a new method of controlled and targeted delivery of vaccine, to directly contact thousands of APCs residing within the skin, without widespread cell death – and exploiting the network of epidermal and dermal APCs [16] to significantly improve vaccine potency over existing delivery approaches.

**Figure 2:**
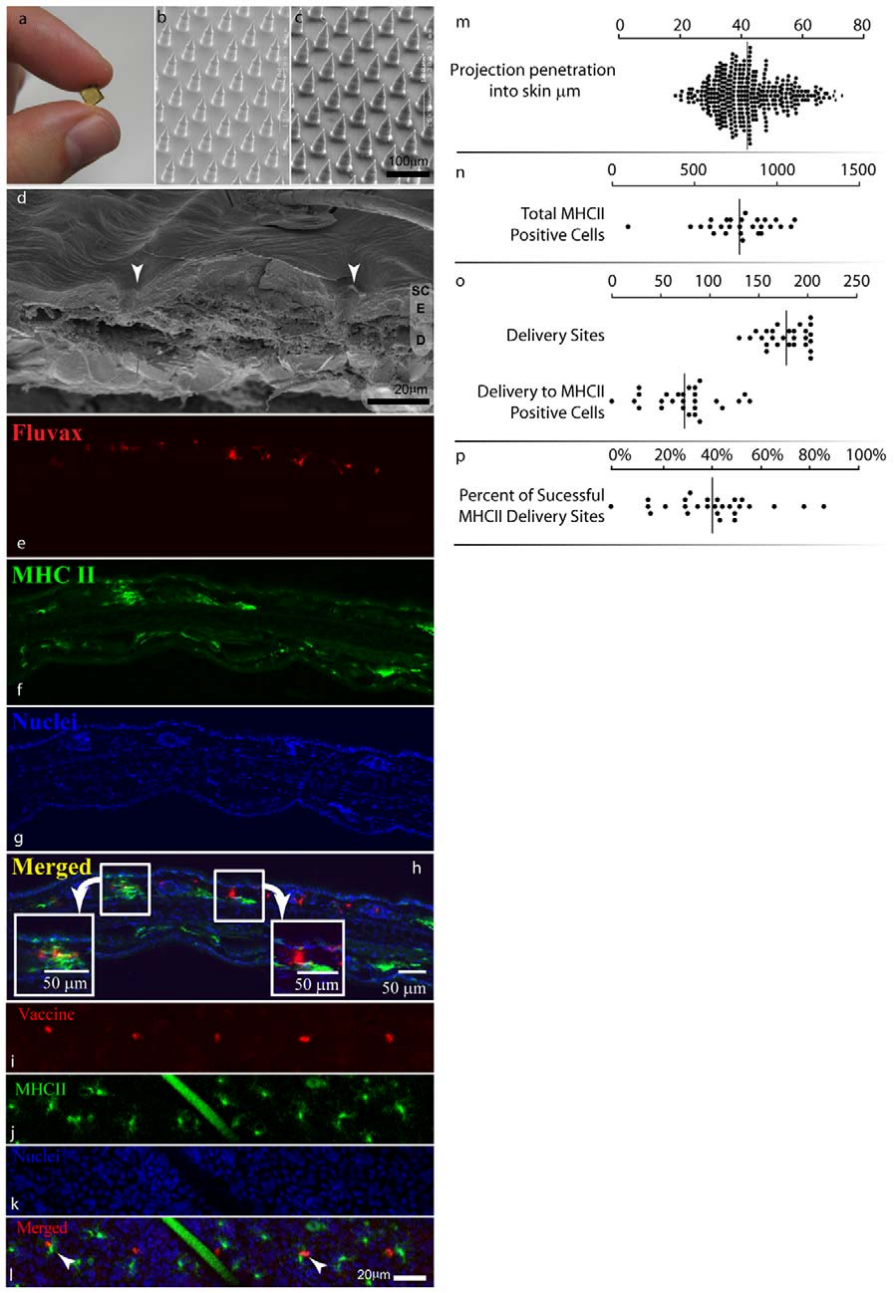
Nanopatch™ appearance, application, and targeted delivery of antigen to antigen presenting cells (APCs). Nanopatches (**a**) were fabricated (cone length 40 µm) with Deep Reactive Ion Etching and then visualized by scanning electron microscopy (SEM) uncoated (**b**) or dry coated with Fluvax®2008 and methylcellulose (**c**). The skin following Nanopatch™ application was visualized using Cryo-SEM (**d**), the arrows show entry points through the epidermis (SC: stratum corneum; E: viable epidermis; D: dermis). The localization of vaccine (red, panel **e side view, panel i top view**; Cy3 labeled Fluvax®) and MHC class II +ve cells (green, **f side view, panel j top view**) was determined by confocal microscopy to the depth of 46 µm. A hair can be seen in **j** and **l** as a large diagonal bar. Nuclear staining (blue, panel **g and k**; stained with Hoechst 33342) in the epidermis was used to determine the epidermal-dermal boundary. Co-localization is shown in panel **h and l** (arrow heads). The distal tip penetration values are shown in the panel **m.** Quantification of MHC class II +ve cells, antigen and co-localized antigen and MHC class II +ve cells was carried out using data from 9 areas within each of three treated ears (**n,o,p** ). An example of a confocal microscopy image in three dimensions is presented in **Video S1**.

To achieve this goal, we designed a Nanopatch™ device (shown conceptually in **Fig 1d**; and **Fig 2 a–c**), displaying an array of densely-packed (21025 projections/cm^2^) gold-coated silicon projections (∼110 µm in total length, including a cone of 40 µm height; tapering to tip designs at <1000 nm) coated with vaccine antigen in *dry form* (**Fig 1 d** **and** **2 c**). When applied to the skin, the concept is for the Nanopatch™ projections to penetrate the epidermis and upper dermis, depositing antigen directly to high populations of APCs residing within these skin layers (**Fig 1 d**). However, we also design the slender projections at the micro-nanoscale (with much of the length below the diameter of target cells), so that when they are slowly applied to the skin (i.e. around 1 m/s, which is much lower than the very high gene gun speeds), they induce low stresses to the target cells, and thus are likely to induce a low incidence of cell death near the tips [17], [18]. In doing so, we are combining the benefits of a more accurately directed delivery of vaccines to a high population of APCs, without widespread cell death. As shown to scale in **Fig 1**, Nanopatches are distinct from existing microneedle devices, by having very high packing density of projections (>20000 projections/cm^2^) tailored by a probability analysis to deposit antigen directly to thousands of epidermal and dermal APCs mapped within the skin, with the smaller diameter far less likely to damage cells near the projections.

In this study, we tested the ability of Nanopatches to deliver seasonal influenza vaccine with delivered dose reduction. We chose commercially-available seasonal human trivalent influenza vaccine (Fluvax® 2008) as a test case, because an antigen-efficient vaccine delivery system for influenza might overcome seasonal vaccine shortages and allow a greater number of people to be vaccinated quickly following the arrival of a pandemic strain [19], [20]. In the event of a pandemic, the current influenza vaccine production capacity of about 900 million doses [20] falls far short of the global population; most of whom might need the vaccine. Conceptually, however, we believe the Nanopatch is broadly applicable to many other vaccines.

## Results

### A probabilistically guided approach for directly depositing antigen to more than 50% of both epidermal and dermal APCs, beneath the patch area

As our starting point we chose Langerhans cells, a key antigen-presenting cell in the skin [16], as just one example target because their depth and location are very well quantified [21]. First, we determined the probability of a single projection contacting a Langerhans cell:
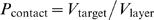
(1)Where:

*V*_layer_ is the volume of the tissue layer containing the Langerhans cells,

*V*_target_ the volume, including the target to which the vaccine can be delivered.

In solving (1), we applied the knowledge of Langerhans density and location: *e.g.* the mouse ear (C57BL/6 Strain) has 542±17 Langerhans cells per square mm of skin [22], tightly distributed in the vertical plane at a mean depth of 14.9 µm below the skin surface for the mouse ear (in humans the density of these cells is similar, but they are deeper), less than 3 µm above the dermo-epidermal boundary and with a normal histogram distribution [21]. We calculated that the chance of a tapering projection contacting a Langerhans cell body was *P*_contact_ = 0.275. This probability calculation is simplified and therefore conservatively low: we assume that the contact event will be an antigen touching the Langerhans cell body, and have not included the other contact events with the network of dendrites extending from the Langerhans cell. With each Langerhans cell possessing between five and nine dendrites – some measuring twice as long as the cell body – this difference is significant (e.g. overall, Langerhans cells can cover a quarter of the total projected skin surface area, while their bodies alone cover just ∼5%) [23]. Therefore, if all the projections penetrate the skin to the Langerhans cell layer, 6728 projections (i.e. using two Nanopatches; 3364 projections per Nanopatch™ covering 0.16 cm^2^ of skin) will accordingly target the bodies of 2000 Langerhans cells – or, when we also take into account the dendrites, about 10000 Langerhans cells (of an available 17350 resident under the Nanopatch™).

Penetrating deeper into the dermis will also target vaccine directly to other classes of APCs [24], that have a less tightly-defined location [25]. Using the knowledge of there being a third of dermal APCs – compared to Langerhans cells in the epidermis – their location, and shorter dendrites [25] we again applied our probability analysis to estimate that our Nanopatch™ designs would target the bodies of approximately 500 dermal APCs, or about 3700 APCs (of an available 7360 residing in the dermis) when we also take into account contact with the cell dendrite arms. In summary, our Nanopatch™ design for this experiment was theoretically configured to deliver antigen directly to more than 50% of both the epidermal and dermal APCs available under the patch area within the skin – without relying on antigen diffusing away from the projections.

### Translating theoretical skin APC targeting into a practical Nanopatch™ design

Guided by our theoretical probability-based analysis, we designed devices with arrays of densely-packed silicon projections (each ∼110 µm in length; with a cone height of 40 µm, at a density of 21025/cm^2^; distributed in 4×4 mm arrays): also designed to be slender enough to puncture individual cells without inducing significant cell death, but at the same time mechanically strong enough to be pushed through the stratum corneum without breaking. To achieve this balance, the projection tips for this experiment were designed to be sharp, tapering down to <1000 nm at the tips, which, when applied to skin at low speeds (∼1 m/s), were predicted to induce minimal cell death [17], [18]. We designed the spacing between the projections to be sufficiently close for the Nanopatches to be practically sized for skin application; while providing enough space for the skin to deflect and compress around the projections during penetration. We fabricated Nanopatches from silicon wafers with Deep Reactive Ion Etching – a method typically used in industry and potentially suitable to high volume manufacture at low unit cost [26].

### Nanopatch™ projections consistently penetrate the skin surface, through to the upper dermis

We then applied the Nanopatches to the skin, first with the goal of establishing the desired consistent and repeatable projection penetration through the epidermis and into the upper dermis.

First, using scanning electron microscopy images of the skin surface following application, we observed 93±2.9% (mean ± SD; n = 350 projections) of the projections penetrated the skin. These data show consistently, that most of projections breach the skin barrier.

We then progressed to measure how far the projections penetrate into the skin. Using the delivery of lipophilic dye by the Nanopatch™ (fluorescent DiD; imaged by confocal microscopy), **Fig. 2m** shows we measured the resultant distal tip penetration depth of 42±10 µm (mean ± SD; n = 350 projections, 5 ears) – extending through the epidermis (17.1 µm in thickness [21]) and into the upper dermis by 25 µm. And in separate experiments, we observed the *in situ* projection track pathways (i.e. the ‘holes’ left behind in the skin by projections; not reliant on dyes; obtained with cryo-scanning electron microscopy (SEM) in which, C57BL/6 mouse ear skin was frozen when the patch was in place). **Fig. 2d** shows a representative projection track pathway piercing fully through the stratum corneum and epidermis – and into the upper dermis. These qualitative observations are consistent with the quantitative lipophilic dye measurements.

### Influenza antigen is directly deposited to thousands of epidermal and dermal APCs, in close accordance with probability-based design

Having established the Nanopatch™ projections achieve the desired penetration into skin, we then measured the resultant delivery of influenza vaccine directly to skin APCs – in practice. Each Nanopatch™ was coated with commercially-available trivalent influenza vaccine (Fluvax®2008; CSL Ltd, Parkville, Australia) and applied to the inner earlobe of anaesthetized female 6–8 week old C57BL/6 mice.

Using immunofluorescence microscopy (**Fig 2e–h** **side view and 2i–l top view**); and, in three dimensions, (**Video S1**), we found that 40±19% of all the projections directly deposited antigen in contact with epidermal and upper dermal MHC class II positive cells. We also examined the skin area subjected to Nanopatch™ application and found the Nanopatches directly targeted antigen to 9.2±5.2% of the cell bodies of resident MHC class II cells imaged within the ‘targeting zone’ (i.e. the skin area the Nanopatch™ is applied to) of epidermis and upper dermis. We measured the overall density of MHC class II cells at 772±212 cells/mm^2^, which is nearly identical with the sum of the Langerhans cells and dermal APCs previously measured [25].

We then considered the population of MHC class II cells are the Langerhans cells – with a density of 542±17 cells/mm^2^ at the suprabasal location within the mouse ear (same strain and site) [22]. Thus, we derived from experiment that the bodies of approximately 1600 Langerhans cells were directly targeted by applying two of our prototype Nanopatches to each mouse. This result agrees approximately with our original theoretical expectation of Nanopatches targeting 2000 Langerhans cell bodies; when we also take into account measurements showing that about 93% of the Nanopatch™ projections penetrate the skin; then our theoretical targeted Langerhans cell body population becomes 1870 cells – even closer to the observed result in practice. We have shown that Nanopatches target vaccine to the desired skin APCs, in populations agreeing well with our original probability-based prediction used to guide the device design. When we also take into account the dendrites extending from these cell bodies, the number of APCs directly targeted by antigen is significantly greater. For example, for Langerhans cells, this could be greater than 9000 of the approximately 17000 resident Langerhans cells in skin under contact of both Nanopatches (0.32 cm^2^).

Looking then deeper into the skin at the dermal APCs – within the same skin area under the Nanopatches – we determined by experiment that about 680 cell bodies were targeted (with further analysis of **Fig 2i–l** ) which is higher than the theoretical calculation of 450 (taking into account 93% of the projections penetrate the skin). When we theoretically also account for the dendrites, then we could have targeted antigen directly to as many as 5000 of the total of approximately 7360 dermal APCs.

Therefore, we have confirmed by experiment that antigen was directly delivered to more than half of the APCs residing in both the epidermal and dermis – also agreeing very well with our probability-based analysis guiding our Nanopatch™ design.

### Nanopatch™ vaccination of mice yields equivalent total anti-influenza IgG responses as intramuscular injection, but with more than 100-fold less antigen delivered to the skin

**Figure 3:**
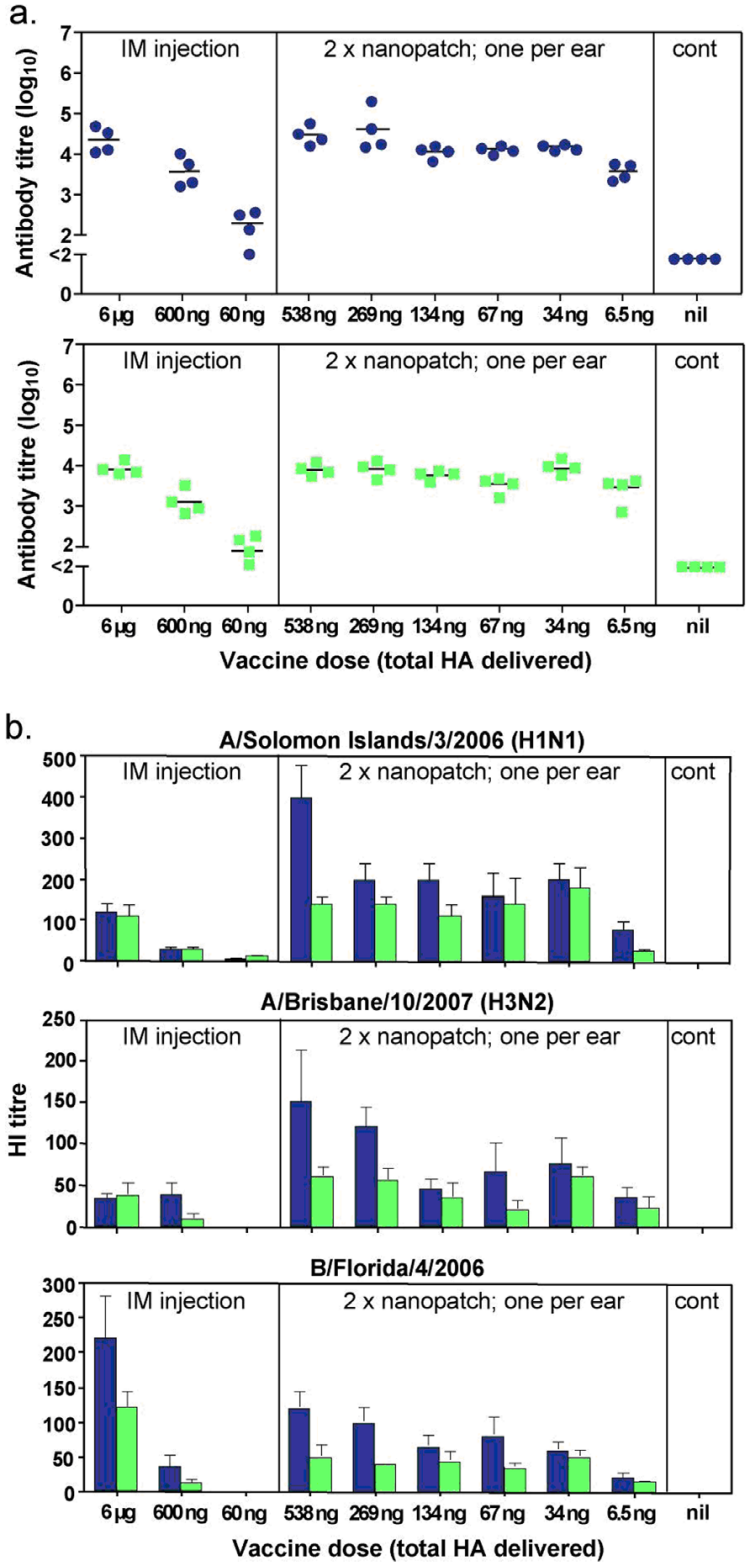
Immune responses induced by influenza vaccine delivered by Nanopatch™ and intramuscular injection. Nanopatches were coated with incremental and controlled doses of the vaccine Fluvax®2008 by the addition of different doses of Fluvax® in the same volume of methylcellulose. The doses indicated are the total hemagglutinin amounts of the three viral strains included in the vaccine. Seven microliter of the coating solution, containing Fluvax® and methylcellulose was applied on each Nanopatch™ followed by drying with a stream of nitrogen gas. Delivered doses were determined by Coomassie Blue R-250 dye recovery. C57BL/6 mice were vaccinated once with one Nanopatch™ on each inner ear lobe or by intramuscular injection with the indicated doses and were bled at 3 weeks and again at 2 months. (**a**,) Individual serum samples (3 weeks, blue circles; 2 months, Green squares) were assayed by ELISA on Fluvax®-coated wells and IgG titers for each individual animal are shown as a closed circle with the geometric mean titer (GMT) as a line. (**b**,) Sera (3 weeks, blue bars; 2 months, green bars) were assayed for hemagglutinin inhibition (HI) activity^38^ against each of the three component viruses included in the trivalent vaccine as indicated above the panels. Bars are the GMT and error bars are the standard error of the mean.

Mice were vaccinated once by applying Nanopatches coated with Fluvax® to both ears and a range of doses were tested across different experimental groups. After application, each Nanopatch™ was kept in place on the skin for 2 minutes, to give adequate time for the vaccine to dissolve and diffuse into the skin subsurface (**Supporting Document Supplementaty Figure S1**). Additional groups of mice were vaccinated by needle and syringe in the caudal thigh muscle. Following this single vaccination, all mice were bled at 3 weeks and also at 2 months. Serum antibodies (IgG) to vaccine antigens were assessed by ELISA [27] (**Fig 3a**). First, considering the 3 week bleed data (**Fig 3a** **, upper panel**) in needle and syringe vaccinated mice, reducing the dose by 10-fold gave a significantly lower response (p = 0.029, Mann-Whitney U test), and by a further 10-fold gave a weak but measureable response in only 3 of 4 animals. In contrast, antigen delivered to the skin using the Nanopatch™ gave antibody responses even at the lowest measured delivered dose tested (6.5 ng), and with a dose of 34 ng showing no significant difference from the 6000 ng intramuscular dose (p = 0.11). This indicates that equivalent high antibody responses can be obtained with the Nanopatch™ compared to intramuscular injection with more than 100-fold less antigen delivered to the skin. These responses persisted at high levels for at least 2 months (**Fig 3a** **, lower panel**).

### More than a 100-fold reduction of delivered antigen dose was observed for functionally relevant antibody (HI) responses, for three strains, when the vaccine was administered using the Nanopatch™

We then examined the functional relevance of the antibody produced in protection against the influenza virus. To do this, we tested the same sera against each of the three vaccine component strains of virus for hemagglutination-inhibitory (HI) activity (**Fig. 3b**). The HI activity assay is the most widely accepted ‘gold standard’ used as the surrogate for influenza protective effectiveness [28], [29].

First, we consider the H1N1 component of the Fluvax 2008®. We see, by three weeks after vaccination, that all the Nanopatch™ vaccinated groups – with delivered doses ranging from 538 ng down to 34 ng – generated equivalent or higher HI titers than 6000 ng intramuscularly vaccinated group. At the lowest Nanopatch™ dose of just 6.5 ng, the HI titer was below responses generated by a 6000 ng intramuscular injection, but still greater than 600 ng intramuscularly vaccinated mice. Then, at two months after vaccination, the comparison between the Nanopatch™ and intramuscular groups yielded the same outcome.

Second, turning to the H3N2 component of Fluvax 2008®, we observe that in all doses the Nanopatch™ delivery (spanning from 538 ng right down to 6.5 ng) induced equivalent or higher HI titers compared to 6000 ng intramuscular injection, at three weeks following vaccination. Following this, at 2 months, we again see significant HI activity maintained in the Nanopatch™ vaccinated mice compared to the intramuscular injected groups. With further examination of the H3N2 component, we see that while none of the intramuscular injections did achieve an HI titer of 1∶40 (even with the highest dose) – which is the minimum level sufficient to correlate with protection in humans [30] ; the Nanopatch™-delivered vaccine yielded HI titers generally above this critical level.

Third, considering the type B virus component of the Fluvax 2008®, we see Nanopatch™ delivery of low doses of HA induced substantial HI activity above the critical value of 1∶40 at 3 weeks, again indicative of protection at doses as low as 34 ng compared to 6000 ng with intramuscular injection. Furthermore, the antibody levels were maintained after 2 months above this critical level in all doses that were 34 ng or higher.

Collectively for each of the three strains, more than a 100-fold reduction of delivered antigen dose was observed for HI responses when the vaccine was administered using the Nanopatch™ – a similar magnitude of improvement as was observed from the IgG antibody responses in the ELISA.

### Delivering an influenza vaccine by Nanopatch™ induces a protective immune response against viral challenge

**Figure 4:**
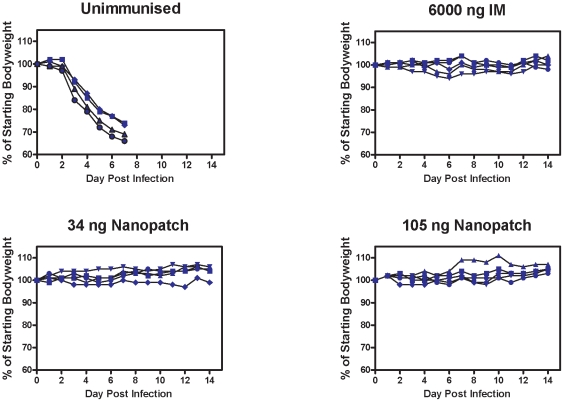
Influenza challenge protection study using PR8 strain. Groups of five C57BL/6 mice were vaccinated with either PBS or different doses of a preparation of split virus vaccine comprising mouse-adapted influenza virus A/Puerto Rico 8/34. Mice were given the vaccine at the doses of 34 ng and 105 ng HA in the form of two Nanopatches to the ears, or with a dose of 6000 ng HA by IM injection with needle and syringe. Thirty six days after the vaccination, mice were challenged with 200 plaque forming units of virus (corresponding to approx. 4 times the lethal dose). The body weights of mice were recorded daily after the challenge. Mice were euthanized at a previously determined humane endpoint indicative of severe illness that would otherwise progress to death.

Although the HI assay is a widely accepted correlate for vaccination protection against influenza virus infection (discussed in many publications, with [28], [29] as two examples), we decided also to measure whether our Nanopatch™-delivered vaccine induced a protective response against influenza virus challenge. To carry out this part of the study, we could not use the commercial trivalent vaccine (Fluvax® 2008) because mice cannot be infected with these human strains of influenza. Therefore, as an alternative, we used a split virion vaccine preparation, based on the mouse-adapted A/Puerto Rico 8/34 strain [31] for use in the protection assays (described in Methods). We see in **Fig. 4** that delivering the vaccine by Nanopatch™ – even with delivered doses as low as 34 ng – induces a protective immune response, as mice exhibited no substantial weight loss or clinical signs of diseases. In contrast all unvaccinated mice were euthanized on day 7 post infection at the humane end-point after dramatic weight loss.

## Discussion

In this study we demonstrated that the Nanopatches deliver influenza vaccine to directly contact thousands of skin APCs according to design, inducing functionally protective immune responses in mice comparable to the vaccine delivered intramuscularly with the needle and syringe, but with less than 1/100^th^ of the delivered dose. This result is an order of magnitude higher than has been previously reported with other delivery devices with only one vaccination, without using adjuvants (and thus, mitigating risk of adjuvant induced adverse reactions) [6]. This significant reduction in the amount of antigen needed to induce an immune response is most likely the result of directly targeting vaccine to a higher proportion of viable APCs in skin (*e.g.* achieved targeting of several thousands of Langerhans cells and dermal APCs per 0.32 cm^2^ of targeted skin) than current delivery methods.

Although it is considered important to target antigen to APCs, it is not known how many cells should be targeted for effective or optimized vaccination. This is the case for both mice and humans. Therefore it follows that the thresholds for eliciting protective immune responses in both mice and humans is also not known. In this paper, we uniquely contribute to the body of knowledge by: (a). Using a probabilistically guided approach to directly target mice to more than 50% of both epidermal and dermal APCs (beneath the patch area). (b). Demonstrating by experiment in mice that antigen is directly deposited to thousands of epidermal and dermal APCs (the bodies of 1600 LC, up to 9000 when we consider the dendrites; to bodies of 680 dermal APC, up to 5000 when we consider the dendrites; respectively), in close accordance with probability-based design. (c). Showing that vaccination of mice yields equivalent total anti-influenza IgG responses as intramuscular injection, but with more than 100-fold less antigen delivered to the skin.

Since a protective response has been elicited in this study, obviously the cells targeted have been above the threshold. Our work in this paper paves the way to finding this threshold in future work. We have now established a rational design and engineering approach for designing Nanopatches to target discrete and incremental ranges of APC populations (both epidermal and dermal). This will be applied in follow-up studies on mice and on humans.

Furthermore, we have shown the actual number and location of targeted APCs – for both the epidermis and dermis – agrees with the probability-based prediction used to guide the device design. This is important, because it demonstrates that a mathematical/engineering design approach ‘from the ground up’ does indeed achieve the desired APC targeting – thereby putting in place a rigorous template for further extending this uniquely precise delivery approach to targeting vaccines to selectively different quantities of APCs. However, these studies have only been performed with one Nanopatch™ configuration and we believe there is much scope to apply this device, together with it's underlying probability-based design approach, as a toolbox for further studies optimizing many vaccines – including those for influenza.

We foresee, most importantly, that our study positions the Nanopatch™ for extension beyond mice and into humans. In doing this, we will again apply the same idea of tailoring the Nanopatch™ device to directly target vaccine to large populations of the epidermal and dermal cells within human skin – accounting for the physiological differences between the skin of the two species. We expect the spatial targeting considerations to be similar, because the density of the characterized APCs resident in the skin is comparable (e.g. Langerhans cell density is 542±17 mm^−2^ in mouse ear skin (for the strain tested in this study) [22]; and 730±60 mm^−2^ in human upper arm skin [32]). However, the human skin is thicker than mouse skin (e.g., mouse ear epidermis is 17.5±2.1 µm [21]; while a candidate vaccination site for humans is the forearm, with a dorsal epidermis is 61.3±11.0 µm [33]). And correspondingly, the depth of Langerhans cell locations (for example), in the supra-basal region, is greater in humans. Reaching this human target, together with underlying dermal APCs, will require deeper penetration of the projections, which could be achieved by slightly longer projections, and exploiting the skin's viscoelastic behavior (e.g. by altering the strain-rate of Nanopatch™ application).

It is worth noting that we have also engineered Nanopatches as practical delivery devices for potential use in humans. They are produced by a simple manufacturing process with the potential for high volume production. By dry-coating vaccine to the Nanopatch™ tips, we expect to minimize or eliminate the need to refrigerate vaccines, as is currently required for most vaccines. The basis for this expectation is that reformulating liquid vaccines into dry-form micro-particles makes them thermostable [34], and we are applying very similar coating principles with our Nanopatch™. Thermostable vaccines have the potential to remove the need for refrigeration – which particularly burden infrastructure in the developing world and/or a pandemic requiring very rapid transportation. The potential for self-application is an added advantage. Nanopatches are also anticipated to avoid needle-phobia, needle-stick injuries (causing more than 500,000 deaths per year), and cross-contamination (e.g. 21 million cases of hepatitis B transmission annually) associated with the hypodermic syringe [35], [36]. With these collective attributes, Nanopatches should be suitable for general immunization programs for many vaccines, particularly where there is a need to avoid the use of needle and syringe as in vaccinations of infants and children. Nanopatches are safe to use in humans because it is made out of silicon and coated with gold and the microprojections do not break during insertion [37], [38]. In a very unlikely case of breakage (expected to be ≪0.1% of projections, the effects on safety are expected to be minimal because both gold and silicon have been approved as safe by the FDA for skin use [39]. Furthermore gold particles have been used extensively in gene gun DNA immunisations approximately 1mg dose delivered into the skin without any deleterious effects (eg. Phase 1 safety and immune response studies of a DNA vaccine encoding hepatitis B surface antigen delivered by a gene delivery device [40]. Any microprojections remaining in the skin will eventually be sloughed off with the skin.

The cost of a vaccination depends on many factors, including: the delivery device (e.g. needle and syringe, or alternative), the vaccine, the cost of transportation, cold chain and the use of a qualified medical practitioner to administer the vaccine and the disposal cost of the device (and packaging). For example, cold chain can contribute 80% of the cost of vaccination programs in developing countries [41]. Although the cost of the Nanopatch is comparable to the cost of the needle and syringe, we believe the ultimate overall cost of vaccine administration via Nanopatch will be considerably cheaper than the needle and syringe, because the Nanopatch is expected to outcompete the needle and syringe in each of the attributes listed.

We speculate that in the future we could rollout a rapid response to pandemic influenza to a much greater population via highly-immunogenic Nanopatch™-delivered vaccine sent in the mail to individuals for self-administration.

## Materials and Methods

### Ethics Statement

All animal experiments were conducted according to the University of Queensland animal ethics regulations.

### Vaccine used

We used was the seasonal human influenza vaccine, Fluvax2008®, manufactured by CSL Ltd, Parkville Australia which contains 15 µg hemagglutinin (HA) per 0.5ml of each of the three viral strains bearing the surface antigens of A/Brisbane/10/2007 (H_3_N_2_), A/Solomon Islands/3/2006 (H_1_N_1_) and B/Florida/4/2006.

### Coating of Nanopatches

Each Nanopatch™ (0.16 cm^2^) was coated with commercially-available trivalent influenza vaccine (Fluvax2008® CSL Ltd, Parkville Australia) using a nitrogen-jet drying coating method [37]. Briefly, 7 µl of coating solution, containing methylcellulose and the vaccine, was applied to each Nanopatch™. A nitrogen gas jet (6–8 m/s) evenly distributed the solution on the whole Nanopatch™, while simultaneously localizing the vaccine on the projections where it would be delivered to skin.

### Measurement of projection penetration in skin, using fluorescent dye

Four DiD (1,1′-dioctadecyl-3,3,3′,3′-tetramethylindocarbocyanine per-chlorate) coated patches were applied to the inner earlobe of anaesthetized female 6–8 week old C57BL/6 mice (one patch per ear) with a custom spring based applicator device at 1.96 m/s. After application, each Nanopatch™ was kept in place on the skin for 2 minutes. Penetration and release profiles were obtained by examining the cryopreserved sections of skin with multi-photon microscopy (MPM, Zeiss 510 Meta, Germany). To track the delivery of fluorescent chemicals from coated microprojections in the skin, we used MPM to produce a series of images at successive skin depths, and then repeated the procedure at later time points. The projection penetration depth for each projection set was determined by image analysis. The delivery of DiD from 130 projections was measured for each ear with a total of 4 ears analysed (i.e. n = 4). The morphology of projections was examined before and after application to the skin with Scanning Electron Microscopy (SEM, JEOL 6400) to qualitatively confirm the coating and release.

### Observation of projection penetration in skin, using Cyro-SEM

Cryo-SEM was employed to visualise the penetration of projections in skin. A patch was applied to the skin by a custom spring based applicator device at 1.96 m/s. With the projections in place, the skin and patch assembly was frozen in liquid nitrogen for 10 seconds. To avoid condensation accumulation on the skin, the array was removed in a cryo-preparation chamber, under vacuum. Then the skin was cut into pieces in the vacuum chamber and the intersection of the skin was observed under SEM (Phillips XL30) at room temperature. Three patches were also applied onto three mouse ears at a consistent and repeatable velocity, by a custom spring-based applicator device at 1.96 m/s. The number of projections which pierced stratum corneum was counted.

### Histology and immunostaining

Four Cy3 labeled Fluvax® coated patches were applied to the inner earlobe of anaesthetized female 6–8 week old C57BL/6 mice (one patch per ear) with a custom spring based applicator device at 1.96 m/s. After application, each Nanopatch was kept in place on the skin for 2 minutes. Mice were subsequently sacrificed and ears excised and the skin was fixed in 2% paraformaldehyde in 0.1 M phosphate buffer at room temperature for 10 minutes. The tissue was then cryopreserved and then sectioned to 10 µm wide and counterstained with Hoechst 33342. A stock solution of 10 mg/ml Hoechst 33342 in DMSO was prepared. A working solution was made by a 1∶10,000 dilution in TBS for nuclei staining. Tissue sections were treated with the working solution of Hoechst 33342 solution and incubated for 10–20 minutes at room temperature. Finally, the tissue sections were rinsed for 3×15 minutes in TBS. The tissue sections were immunostained with mouse anti-MHC II-FITC (eBioscience, San Diego, CA USA) at 1∶500 for 1 hour at room temperature. The sections were then rinsed for 3×15 minutes in TBS. The tissue was then mounted with Prolong Anti-fade Gold (Invitrogen, Carlsbad, CA USA).

Whole ears were split at the dermis-cartilage junction where the epidermis and dermis from the application side were retained for staining. The split-ear tissue was fixed in 2% paraformaldehyde in 0.1M phosphate buffer at room temperature for 30 minutes. The tissue was then washed 3×15 minutes in TBS prior to permeabilization with 0.1% Triton-X 100 in 0.1M phosphate buffer at room temperature for 15 minutes. The tissue was then washed again 3×15 minutes in TBS. The tissues were immunostained with mouse anti-MHC II-FITC (eBioscience, San Diego, CA USA) at 1∶200 for 1 hour at room temperature. The sections were then rinsed for 3×15 minutes in TBS. The tissue was then mounted with Prolong Anti-fade Gold (Invitrogen, Carlsbad, CA USA).

We have defined the targeting of antigen directly to an APC as:

- the antigen co-localized with the APC (i.e. the color yellow), and/or
- the antigen in direct contact with the APC. We define this contact as there not being any ‘empty’ voxels between the fluorescent signals from the antigen and the APC. The voxel size is 0.82 µm × 0.82 µm × 1.8 µm (x, y, z), defining the resolution of this direct contact event.

### Use of Coomassie Blue R-250 as the tracer dye to determine the vaccine dose delivered

We measured the dose of Fluvax® antigen delivered to the skin using Coomassie Blue R 250 dye as a tracer. Correspondingly, the mass of delivered Fluvax® antigen was determined by the ratio of Fluvax® to Coomassie Blue R-250 contained in the coating solution. Five Nanopatches were coated with Fluvax 2008®, Coomassie Blue R-250 dye and methylcellulose. With the same applicators, the coated Nanopatches were applied to mouse ears (n = 5). Then the skin sites were gently and thoroughly cleaned with a cotton swab moistened with physiological saline immediately following patch removal. Subsequently, the skin from the patched area was cut and then homogenized in 1 ml of 70% ethanol at 55°C for 2 hours under stirring to extract the Coomassie Blue in the skin. Finally the absorbance (at λ_max_ = 592 nm) of the eluate for the 5 samples was measured and the percentage of Coomassie Blue delivered to the skin from each Nanopatch™ was calculated to be 6.46%±0.84% (Mean±SEM) of Coomassie Blue applied to each mouse ear. Using scanning electron microscopy images of coated Nanopatches, we then examined the coating morphology, and found the coating thickness was uniform across a given Nanopatch™ and repeatable between Nanopatches. This, for example, is demonstrated by the uniform coating thickness at the middle of the cylindrical part of projections (2.10±0.18 µm; n = 5). Furthermore, uniformity of coating to projections was independently confirmed by consistent fluorescence intensity on the projections – measured by confocal microscopy images of Nanopatches coated with a fluorescent dye (rhodamine-dextran; used as a surrogate of vaccine for coating). This collective work validated the assumption that a consistent and repeatable portion of Fluvax® antigen was delivered to skin by each coated Nanopatch™.

### Vaccination of mice

C57BL/6 female mice (groups of five) were vaccinated once by applying Nanopatches dry coated with Fluvax 2008® to the inner lobe of each ear (two Nanopatches per mouse) and held in place for 2 min for the vaccine to diffuse into the epidermis/dermis. A range of doses were tested across different experimental groups. Additional groups of mice were vaccinated by needle and syringe in the caudal thigh muscle. The doses shown are the total HA amounts of the three viral strains combined delivered under the skin of the three different strains present in Fluvax 2008®. Following the single vaccination, all mice were bled at 3 weeks and also at 2 months. The sera were separated and stored frozen at −20°C till assays were performed. All animal experiments were conducted according to the University of Queensland animal ethics regulations.

### Serological analyses

ELISA was performed as previously described [42]. Briefly, the ELISA plates (Nunc Maxisorp) were coated with the commercial trivalent split virion Fluvax2008®. The vaccine was diluted at a concentration equivalent to 3 µg/ml total HA in 0.1M sodium bicarbonate buffer and 50µl of this solution was added to each well of the ELISA plates and incubated overnight at 4°C. The plates were blocked with 4mg/ml BSA and used to determine the titers of antigen specific IgG induced. The color development was performed using ABTS (2,29-azino-bis[3-ethylbenzthiazoline-6-sulfonic acid]) (Sigma cat. no. A-1888) as the substrate. The absorbance readings at 405 nm were measured against control wells containing no antiserum in the reaction. Each sample was individually analyzed.

### Hemagglutination-Inhibition Assay

Sera were treated with receptor destroying enzyme (RDE II, Denka Seiken Co. Ltd.) prior to HI analysis to remove nonspecific inhibitors of agglutination. Samples were diluted 1in 5 in RDE and held at 37°C overnight. An equal volume of sodium citrate 1.6% (w/v) was then added and held at 56°C for 2 hr to neutralize RDE activity. The HI test was performed against each of the three purified influenza viruses present in the Fluvax®2008 formulation using chicken red blood cells by established methods [43] adapted to microtiter format.

### Viral challenge

Mice (n = 5) were vaccinated with a preparation of mouse adapted influenza virus A/Puerto Rico/8/34 split virion [31]. Mice received 34 ng or 105 ng by Nanopatch™ or 6000 ng by intramuscular injection. Unimmunized mice were used as a control. Thirty six days after the vaccination, mice were challenged with a lethal dose of 200 plaque forming units of the virus. Body weights and clinical signs of mice were recorded daily after challenge and, where necessary, mice were culled at a predetermined humane endpoint under guidelines approved by the University of Melbourne Animal Ethics Committee.

### Statistical analyses

ELISA, HI titers and the data in the Fig 2 were analyzed by Mann-Whitney U test using GraphPad Prism 5 software (La Jolla, CA, USA).

## Supporting Information

Figure S1 Figure S1 shows the normalized release of influenza vaccine into skin, following application of Nanopatches bearing dry-coated influenza vaccine to the inner lobe of the mouse ear for different times. The data shows that the released amount of influenza vaccine in the skin does not show statistical difference (p = 0.46) for Nanopatch application time of 2 and 5 minutes. For each application time (0.5, 2 and 5 minutes), a group of 6 coated patches were applied on 6 individual mouse ears for measurement of the released amount of coating in the skin. Video S1. This video shows part of a Nanopatch site in 3D. The Fluvax 2008® payload is red (Fluvax-Cy3) and the antigen presenting cells are shown in green (MHC class II). The image only contains the epidermis and the nuclei are shown in blue (Hoechst 33342). The skin was fixed in paraformaldehyde immediately after the patch was applied. Immunostaining for MHC class II immediately followed fixation. The Z stack video was generated by 3D rendering a z-stack in Imaris. The initial scene is the full image of Figure 2 (i)–(l). The movie then zooms in on a deposit site where the Fluvax co-localizes with an antigen presenting cell.
(0.25 MB TIF)

Video S1 Multimedia File Video S1
(6.02 MB AVI)
