# Supplementary figures and images for: Potent Immunity to Low Doses of Influenza Vaccine by Probabilistic Guided Micro-Targeted Skin Delivery in a Mouse Model
Source: PLoS One. 2010 Apr 21;5(4):e10266. doi: 10.1371/journal.pone.0010266 (PMC2858085; doi:10.1371/journal.pone.0010266)

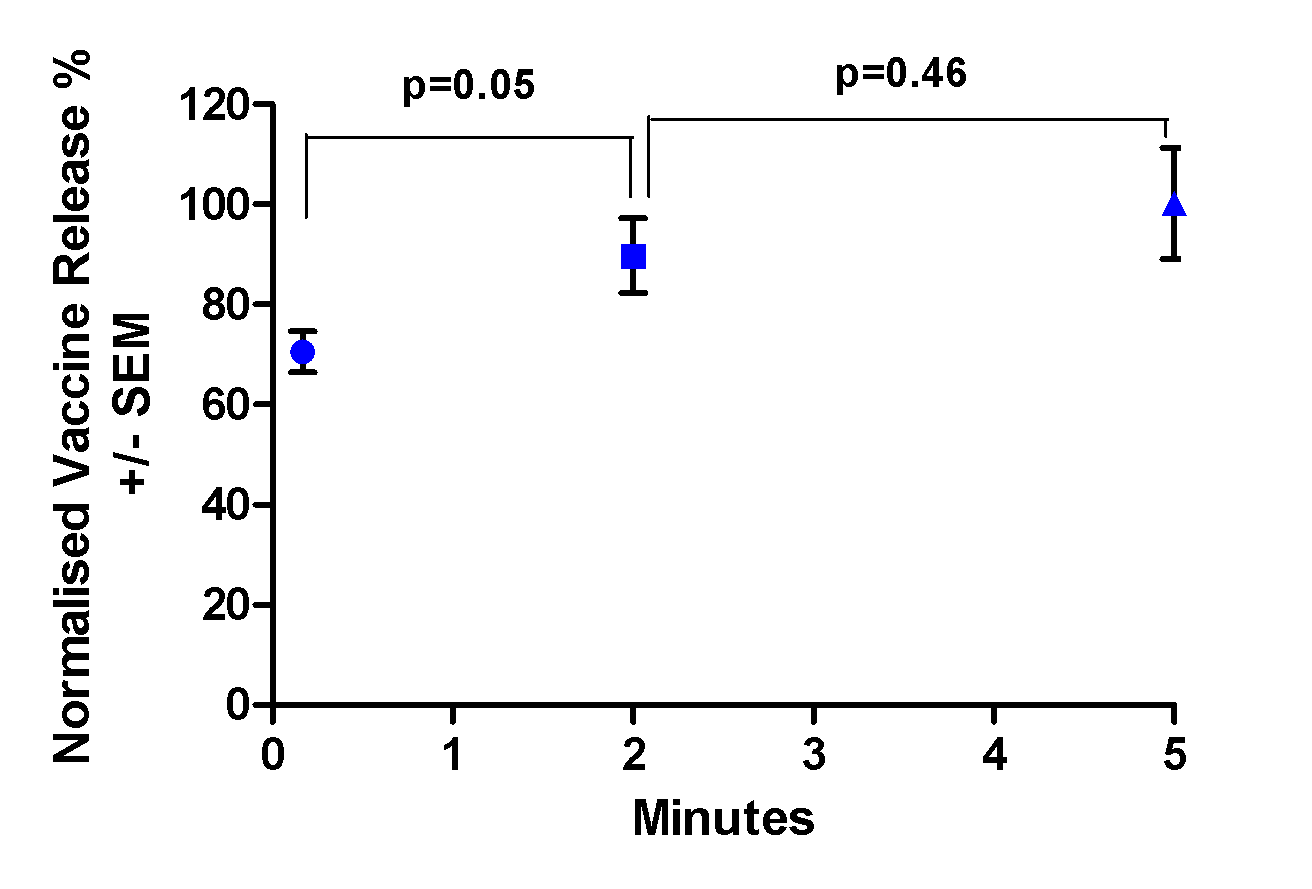

Supplement: Figure S1 — shows the normalized release of influenza vaccine into skin, following application of Nanopatches bearing dry-coated influenza vaccine to the inner lobe of the mouse ear for different times. The data shows that the released amount of influenza vaccine in the skin does not show statistical difference (p = 0.46) for Nanopatch application time of 2 and 5 minutes. For each application time (0.5, 2 and 5 minutes), a group of 6 coated patches were applied on 6 individual mouse ears for measurement of the released amount of coating in the skin. Video S1. This video shows part of a Nanopatch site in 3D. The Fluvax 2008® payload is red (Fluvax-Cy3) and the antigen presenting cells are shown in green (MHC class II). The image only contains the epidermis and the nuclei are shown in blue (Hoechst 33342). The skin was fixed in paraformaldehyde immediately after the patch was applied. Immunostaining for MHC class II immediately followed fixation. The Z stack video was generated by 3D rendering a z-stack in Imaris. The initial scene is the full image of Figure 2 (i)–(l). The movie then zooms in on a deposit site where the Fluvax co-localizes with an antigen presenting cell. (0.25 MB TIF) [file pone.0010266.s001.tif]
